# Introduction the revolving scarf osteotomy for treating severe hallux valgus with an increased distal metatarsal articular angle: a retrospective cohort study

**DOI:** 10.1186/s12891-019-2874-8

**Published:** 2019-11-03

**Authors:** Xinwen Wang, Qian Wen, Yi Li, Cheng Liu, Kai Zhao, Hongmou Zhao, Xiaojun Liang

**Affiliations:** 1 Department of Foot and Ankle Surgery, Honghui Hospital, Xi’an Jiaotong University, 710054, Xi’an, Shan’xi Province China; 2 Physical Examination Center, The Ninth Hospital of Xi’an Affiliated Hospital of Xi’an Jiaotong University, Xi’an, Shaanxi China

**Keywords:** Hallux valgus, Distal metatarsal articular angle, Orthopaedic, Revolving scarf osteotomy;, Double metatarsal osteotomy

## Abstract

**Background:**

Hallux valgus(HV) with an increased distal metatarsal articular angle (DMAA) is one of the most common foot deformities among adults. Double metatarsal osteotomy (DMO) is effective in treating severe HV deformity with an increased DMAA. However, this technique presents the risk of avascular necrosis (AVN) of the metatarsal head and transfer metatarsalgia due to shortening of the first metatarsal. The aim of this study was to introduce a surgical procedure defined as revolving scarf osteotomy (RSO) and compare the clinical and radiological results of RSO and DMO performed for treating severe HV with an increased DMAA.

**Methods:**

First metatarsal osteotomies and Akin osteotomy were performed in 56 patients (62 ft) with severe HV with an increased DMAA in Honghui Hospital from January 2015 to December 2017. RSO was performed in 32 ft and DMO was performed in 30 ft. The Akin osteotomy was performed in both groups. The American Orthopedic Foot and Ankle Society (AOFAS) score, visual analogue scale (VAS) score, the hallux valgus angle (HVA), intermetatarsal angle (IMA), DMAA, and first metatarsal length (FML) and the rates of complications were compared preoperatively and postoperatively in the two groups.

**Results:**

The mean AOFAS score, VAS score, HVA, IMA, and DMAA showed significant improvements in both groups after surgery, but with no significant differences between the two groups. The postoperative FML was significantly larger in the RSO group than in the DMO group (*p* < 0.001). One of the 30 ft (3.3%) in the DMO group exhibited transfer metatarsalgia at 12 months postoperatively, while another foot (3.3%) in same group had avascular necrosis of the metatarsal head. One of the 30 ft (3.1%) in the RSO group had hallux varus.

**Conclusions:**

No differences in the clinical and radiographic results were observed between the two groups with severe HV and an increased DMAA. However, RSO does not cause shortening of the metatarsal and AVN of the metatarsal head. A long-term, randomized, controlled prospective study with a larger sample would provide higher-level evidence for confirming the clinical efficacy and safety of RSO.

## Background

Hallux valgus(HV) manifests as a structural deformity of the first metatarsophalangeal joint with lateral deviation of the great toe and medial deviation of the first metatarsal [1]. HV is one of the most common foot deformities among adults, with a reported prevalence that ranges from 21 to 70% in epidemiological studies [2–7], and is higher in females than in males, and increases with age [8]. HV with an increased distal metatarsal articular angle (DMAA) is more common in adolescents with HV or in patients of any age with a long history of HV [9, 10]. DMAA reflects the matching of the first metatarsophalangeal joint and is a key factor in determining the postoperative recurrence of deformity [11]. Surgical treatment is usually recommended for symptomatic patients with moderate or severe deformity [12]. There is increasing evidence that double metatarsal osteotomy (DMO) is effective in treating severe HV deformity with an increased DMAA [13–15]. However, this technique presents the theoretical risk of avascular necrosis (AVN) of the metatarsal head and transfer metatarsalgia due to shortening of the first metatarsal.

Scarf is a carpentry term describing beveling the ends of 2 pieces of wood and securely fastening them so that they overlap to create one continuous piece. This technique was popularized by Weil and Barouk as a versatile method of correcting hallux valgus while maintaining the blood supply to the metatarsal head [16].. However, scarf osteotomy is not suitable for severe HV deformities with a larger DMAA [17], and so we have created a surgical procedure defined as revolving scarf osteotomy (RSO) to address this treatment deficit. This is a report on RSO for treating HV with increased DMAA. We introduce this method of RSO and compare the clinical and radiographic results between RSO and DMO.

## Methods

### Design

This is a retrospective cohort study. Patients or family members selected for one of the two operations after the doctor introduced the two methods of operation. The study has received approval from the institutional review board of Honghui Hospital(Protocol Number 1809764). The data we collected and analyzed were anonymous, and the requirement for informed consent was therefore waived [18] as Filion, K.B et al. demonstrated.

### Patients

Patients are able to enroll in the study if they meet the following criteria:

Inclusion criteria:

1. Diagnosed with severe HV [intermetatarsal angle (IMA) > 15°or hallux valgus angle (HVA) > 30° [19–21]) with an increased DMAA ≥15°].
2. First received RSO or DMO as the primary HV surgery.

Exclusion criteria:

1. Degenerative osteoarthritis of the first metatarsophalangeal joint, rheumatoid arthritis, neurological diseases, vascular diseases, diabetes mellitus, previous surgery to the front of the foot, or Body Mass Index (BMI) > 30 kg/m2.
2. Incomplete follow-up data.

### Study overview

Fifty-six patients with severe HV with an increased DMAA who presented from January 2015 to December 2017 were selected using the medical record system of Honghui Hospital (Xi’an Jiaotong University) according to the inclusion and exclusion criteria above. The patients were divided into 30 (32 ft) who received RSO and 26 (30 ft) who received DMO. The American Orthopedic Foot and Ankle Society (AOFAS) score, visual analogue scale (VAS) score, the HVA, IMA, DMAA, and first metatarsal length (FML) and the rates of complications were compared preoperatively and postoperatively in the two groups.

### Clinical evaluation

The scores on the hallux metatarsophalangeal interphalangeal scale developed by the American Orthopedic Foot and Ankle Society [22] were used for evaluating the clinical effects. This scale comprises pain (40 points), function (50 points), and alignment (10 points), and does not rely on imaging techniques. A 100-mm-long visual analogue scale was used to measure the perceived pain level [23]. The higher the score, the worse the pain.

### Radiographic evaluations

Measurements of the IMA, HVA, and DMAA performed during hospitalization and follow-up were based on a standardized weight-bearing anteroposterior radiograph of the foot. The HVA was defined as the angle between the line from the center of the metatarsal base to the center of the first metatarsal head, and the line connecting the midpoints of the proximal and distal articular surfaces of the proximal phalanx. The IMA was defined as the angle between the line that connects the center of the base and head of the first metatarsal, and the line bisecting the diaphyseal portions of the second metatarsal [24, 25]. The DMAA is the angle between the first metatarsal axis and the distal articular surface of the first metatarsal [26]. The length of the first metatarsal was measured using the method of Munuera et al. [27] and Nakagawa et al. [28] as the distance between the distal ends of the head and base of the metatarsal.

All measurements were based on a radiograph of the foot in the medical records and they were performed by a trained foot and ankle surgeon who was not involved in the surgeries of the present study in order to avoid both bias and interobserver variability. The data were measured twice and then took the average as the statistical data.

### Operative protocol

General anesthesia combined with lower limb nerve block was applied to all patients in the supine position. The leg was exsanguinated with an elastic bandage, and a tourniquet was applied to the proximal thigh. The first metatarsophalangeal joint and the proximal end of the first metatarsal were exposed by making a medial metatarsal incision. Any bunion present was removed using this incision technique. A dorsal incision was made between the first and second intermetatarsal spaces and the adductor hallucis muscle, lateral collateral ligament, and capsule with lateral sesamoid suspensory ligament were released.

**Fig. 1:**
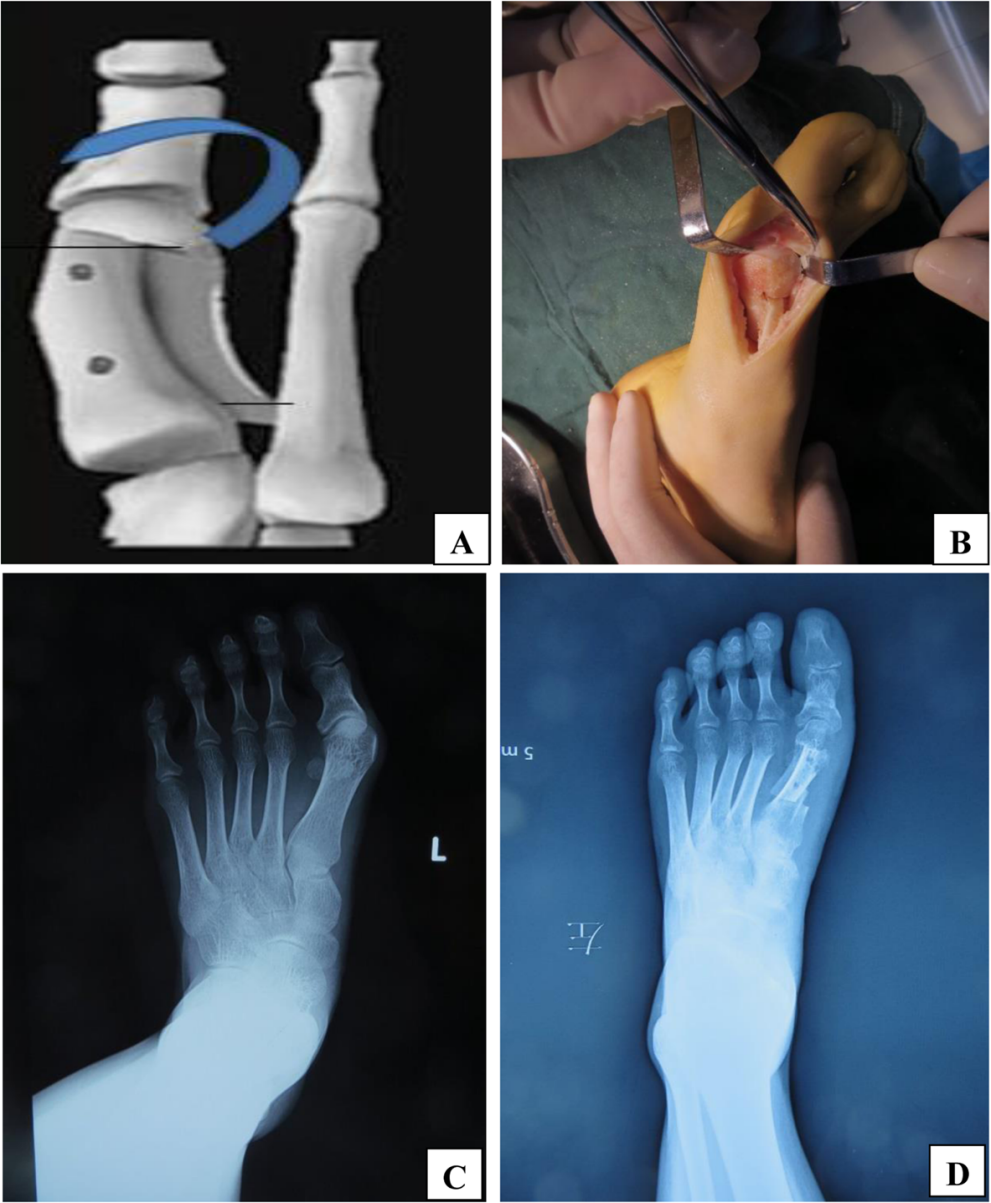
Operative schematic diagram and preoperative and postoperative X-rays. **a**: The diagram of Revolving Scarf Osteotomy **b**: the rotated articular surface during the operation **c**: preoperative antero-posterior **d**: postoperative anteroposterior weight bearing X-rays of a patient who has undergone revolving scarf osteotomy

In the RSO group, a ‘Z’ osteotomy was performed on the medial side of the first metatarsal. Two wedge-shaped sites were then removed from the distal and proximal ends. The distal osteotomy site was pushed outward to correct the IMA. At the same time, the distal bone block was rotated in the reverse direction so as to maintain the position of the metatarsal head; this reverse rotation could correct the DMAA. The osteotomy was then fixed with two screws (Fig. 1).

**Fig. 2:**
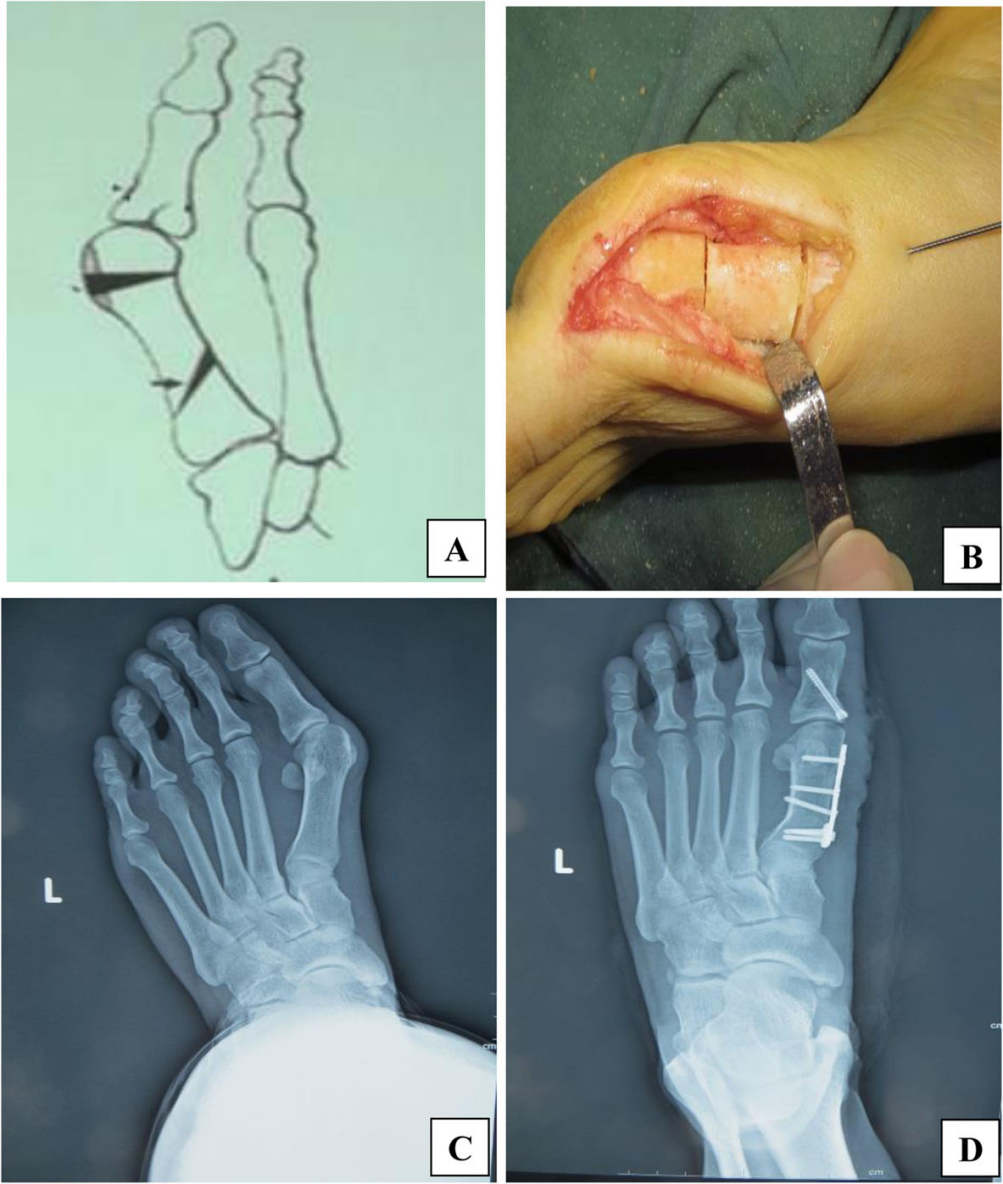
Operative schematic diagram and preoperative and postoperative X-rays. **a**: The diagram of Double Metatarsal Osteotomy **b**: Proximal and distal osteotomy lines during the operation **c**: Preoperative antero-posterior **d**: postoperative anteroposterior weight bearing X-rays of a patient who has undergone Double Metatarsal Osteotomy

In the DMO group, a wedge osteotomy was performed on the first proximal metatarsal and the IMA was corrected in accordance with the method described by Park et al. [29]. A Reverdin osteotomy was performed at the head and neck of the first metatarsal bone to correct the DMAA. The two osteotomy lines were then fixed with a microplate (Fig. 2).

Finally, the Akin osteotomy was performed in both groups, and the medial joint capsule was closed using absorbable sutures.

### Postoperative care

The same postoperative regime was applied in both groups. Compressive elastic bandages were applied after surgery, and gauze was used to isolate the great and second toes. The dressing was changed the next day. At 2 weeks after the operation, the feet were raised, the toes could be moved actively, and the wound thread was removed. Passive movements of the metatarsophalangeal joint had gradually strengthened at 3 weeks after the operation, and an X-ray examination was performed after 6 weeks. If the osteotomy line had healed, the patients were allowed to wear shoes for weight-bearing.

### Statistical analysis

Mean ± Standard Deviation (SD) values were used to express continuous variables conforming to a normal distribution. The difference between preoperative and postoperative values was normally distributed, and so a paired-samples -test was used to compare these values. Wilcoxon’s signed-rank test was applied to other variables. The Shapiro-Wilk test was used to test whether the data conformed to a normal distribution. An independent-samples t-test was used to compare differences between the two groups. Enumerated variables were expressed as ratios. The hypothesis was tested by Pearson’s chi-square test and the Fisher-Freeman-Halton test.

All statistical analyses were performed using SPSS (version 21.0). Statistical significance set at *P* < 0.05.

## Results

The patient characteristics including age, sex, AOFAS score, VAS score, HVA, IMA, DMAA, and FML at baseline did not differ between the two groups (all *p* > 0.05). The prevalence of severe HV with an increased DMAA was higher in females than in males (Table 1).

**Table 1:** Patient characteristics at baseline

characteristic	RSO *n* = 32 ft	DMO *n* = 30 ft	*p* Value
Age (y)	47.09 ± 10.30	47.16 ± 9.48	0.97
Female gender (%)	31(96.8%)	29 (96.7%)	0.738
Preoperative AOFAS score	57.69 ± 5.83	56.60 ± 6.38	0.467
Preoperative VAS score	6.53 ± 1.67	6.67 ± 1.24	0.719
Preoperative HVA (°)	40.5 ± 2.59	40.53 ± 3.01	0.963
Preoperative IMA (°)	14.47 ± 3.80	15.37 ± 3.49	0.337
Preoperative DMAA (°)	30.75 ± 5.14	29.83 ± 5.65	0.506
Preoperative FML (mm)	54.91 ± 3.28	54.97 ± 3.01	0.94

AOFAS (American Orthopedic Foot and Ankle Society) VAS (visual analogue scale) HVA (Hallux Valgus Angle) IMA (Intermetatarsal Angle) DMAA (Distal Metatarsal Articular Angle) FML (First Metatarsal Length) RSO (Revolving Scarf Osteotomy) DMO (Double Metatarsal Osteotomy) Female gender is represented by proportion, and Fisher-Freeman-Halton test was used. The other indices are represented by Mean ± SD, and independent-samples t-test was used

In the RSO group, the AOFAS score increased from 57.69 preoperatively to 89.22 at the first year after surgery (*p* < 0.001), while the VAS score reduced from 6.53 to 2.19 (p < 0.001). The same changes in the AOFAS and VAS scores occurred in the DMO group, and there were no significant between-groups differences in the postoperative AOFAS and VAS scores (AOFAS scores, *p* = 0.664; VAS scores, *p* = 0.407) (Table 2).

**Table 2:** Clinical parameters of two group

Clinical parameters	RSO *n* = 32	DMO *n* = 30	*p*
AOFAS Score			
Preoperative	57.69 ± 5.83	56.6 ± 5.85	0.467
12 months postoperative	89.22 ± 5.96	88.53 ± 6.38	0.664
change	−31.53 ± 5.95	−31.93 ± 6.67	0.803
P_1_	< 0.001	< 0.001	
VAS Score			
Preoperative	6.53 ± 1.67	6.67 ± 1.24	0.719
12 months postoperative	2.19 ± 0.93	2.0 ± 0.83	0.407
change	4.34 ± 2.03	4.67 ± 1.52	0.482
P_2_	< 0.001	< 0.001	

Change were calculated as difference values between values and values 12 months postoperatively. (Change = Preoperative – postoperative). AOFAS (American Orthopedic Foot and Ankle Society) VAS (Visual Analogue Scale) *P* values were derived from independent-samples t-test. P1 and P2 values were derived from a paired samples t-test

In the radiological assessment, the postoperative HVA, IMA, and DMAA values were all significantly lower than the preoperative values in both groups (all *p* < 0.001), with no intergroup differences (HVA, *p* = 0.174; IMA, *p* = 0.416; DMAA, *p* = 0.175). The postoperative FML was significantly larger in the RSO group than in the DMO group (p < 0.001) (Table 3).

**Table 3:** Clinical parameters of two group

Radiological parameters	RSO *n* = 32	DMO *n* = 30	*p*
HVA			
Preoperative	40.5° ± 2.59°	40.53° ± 3.01°	0.963
12 months postoperative	10.16° ± 2.80°	11.03° ± 2.16°	0.174
change	30.34° ± 3.39°	29.5 ± 3.79	0.359
*P*	< 0.001	< 0.001	
IMA			
Preoperative	14.47° ± 3.8°	15.37° ± 3.49°	0.337
12 months postoperative	6.25° ± 1.05°	6.03° ± 1.03°	0.416
change	8.23° ± 3.76°	9.33° ± 3.41°	0.227
*P*	< 0.001	< 0.001	
DMAA			
Preoperative	30.75° ± 5.14°	29.83° ± 5.65°	0.506
12 months postoperative	5.97° ± 1.28°	6.47° ± 1.57°	0.175
change	24.78 ± 4.78	23.37° ± 5.29°	0.274
*P*	< 0.001	< 0.001	
First Metatarsal Length (mm)			
Preoperative	54.91 ± 3.28	54.97 ± 301	0.94
12 months postoperative	56.06 ± 2.94	52.23 ± 2.67	< 0.001
change	−1.57 ± 0.81	2.73 ± 1.89	< 0.001
*P*	< 0.001	< 0.001	

Change were calculated as difference values between values and values 12 months postoperatively. HVA (Hallux Valgus Angle) IMA (Intermetatarsal Angle) DMAA (Distal Metatarsal Articular Angle) FML (First Metatarsal Length) RSO (Revolving Scarf Osteotomy) DMO (Double Metatarsal Osteotomy)

One of the 30 ft (3.3%) in the DMO group exhibited transfer metatarsalgia at 12 months postoperatively. This patient did not receive a second operation for metatarsalgia, and was treated with orthotics for pain resolution. Another foot (3.3%) in the DMO group exhibited AVN of the metatarsal head, and the patient received metatarsophalangeal arthrodesis. These complications were not observed in the RSO group. In the RSO group, one of the 30 ft (3.1%) had hallux varus, which was corrected in a second operation (Table 4).

**Table 4:** Complications at 12 months postoperative

	RSO *n* = 32	DMO *n* = 30
Transfer metatarsalgia	0	1 (3.3%)
Hallux varus	1 (3.1%)	0
Avascular necrosis of the metatarsal head	0	1 (3.3%)

## Discussion

DMO is widely used to correct HV in adolescent patients with an increased DMAA, and there have also been some reports of good outcomes after DMO for adolescent HV [30, 31]. However, the outcomes for DMO in adult HV deformities with an increased DMAA are not clear [32]. Moreover, AVN of the metatarsal head and transfer metatarsalgia often occur after DMO; in our study we found two patients with such complications.

We developed a method of RSO that is modified from scarf osteotomy. In this modification, the head of metatarsal bone is rotated in the reverse direction to produce a more efficient correction effect of the DMAA. The following points should be noted during the operation. First, the main point of the osteotomy is to cut off the wedge-shaped bone on the inner side of the distal end, pull the proximal bone inward, and push the distal end outward to correct the IMA. At the same time, the DMAA is corrected by rotating the metatarsal head in the reverse direction so that it matches the first metatarsophalangeal joint. Second, the osteotomy should be completed in a single procedure with an oscillating saw in order to avoid rotation difficulties caused by unevenness of the osteotomy surface. Third, in the longitudinal osteotomy, the tail of the micro-swing saw raised and the osteotomy is performed from the inner top to the outer bottom; otherwise the first metatarsal head may be raised, causing transfer metatarsalgia [33]. Fourth, combining a scarf osteotomy with an Akin osteotomy will not affect the blood supply [34] and so should be recommended. Fifth, the medial cutaneous nerve should be protected due to the long medial incision; otherwise, the incision will be painful for a long time after the operation. Two patients in the present study had medial cutaneous nerve injury of the affected foot, resulting in numbness of the skin on the edge of the medial incision. However, the symptoms were relieved after 10 months.

In our cohort the mean AOFAS score improved from 57.69 at the preoperative assessment to 89.22 at the first-year follow-up. There was also a significant improvement in pain at 12 months postoperatively, with the mean VAS score changing from 6.53 preoperatively to 2.19 postoperatively. DMO has the same clinical effectiveness as our new technique.

Our osteotomy method improved the three main radiographic parameters of HV: HVA, IMA, and DMAA. The IMA and HVA are objective parameters for assessing corrections performed in each type of osteotomy [24, 25], while the DMAA is commonly used to quantify articular deformity [34, 35]. The DMAA is one of the most important radiographic angles in HV, and it has been shown that non-correction of DMAA alterations is associated with early recurrence of the deformity, reduced range of motion of the metatarsophalangeal joint, and pain [36–38], and so correcting the increased DMAA is key to a satisfactory surgical outcome. The postoperative DMAA had improved significantly compared with the preoperative DMAA in both of the present groups. The mean DMAA correction was 23.37° in the DMO group, which is larger than previous reports [13, 30]; this difference is probably due to the patients in our study having larger DMAA. The same correction of the DMAA was observed in the RSO group. In short, our technique provides similar corrections of the HVA, IMA, and DMAA as for DMO even in the presence of more-severe deformities.

The postoperative FML was shorter than the preoperative FML in the DMO group, while it was longer in the RSO group after the operation. It was confirmed that the onset of metatarsalgia is related to shortening of the first metatarsal [34, 39]. In theory, the incidence of transfer metatarsalgia should be more in the DMO group, and we found only one patient with such pain; this incidence (3.3%) is lower than that reported by Park and Lee (8.7%) [15]. Also, partial AVN of the metatarsal head developed in one foot (3.3%) in the DMO group. AVN can occur due to the wide dissection of soft tissue and multiple osteotomies of the first metatarsal required. In particular, the risk of AVN might be greater if lateral soft-tissue release is performed simultaneously [15]. None of the patients in the RSO group exhibited transfer metatarsalgia or AVN, while one (3.1%) patient in that group had hallux varus due to the medial articular capsule being sutured too tightly and the excessive pursuit of seed-bone reduction.

Notwithstanding the positive findings of our study, it also had the following shortcomings: (1) there was no evaluation of the sesamoid position from the viewpoint of weight-bearing or the metatarsophalangeal range of motion, (2) the follow-up period was inadequate for evaluating long-term HV recurrence, and so a longer-term follow-up study is required, (3) the sample was small and came from a single center, (4) the study had the deficiencies inherent in a retrospective design design, and (5) bias may have been present due to the clinical and radiological measurements not being made by an independent assessor.

## Conclusions

We conclude that our method of RSO in combination with the Akin osteotomy is a safe, reliable, and effective procedure for correcting symptomatic severe HV with an increased DMAA. The favorable aspects of this technique that should encourage its use are the rapid bone healing and the reliable orthopedic effects. A long-term, randomized, controlled prospective study with a larger sample would provide higher-level evidence for confirming the clinical efficacy and safety of RSO.

## Data Availability

The datasets used and/or analyzed during the current study are available from the corresponding author on reasonable request.

## Abbreviations

AOFAS: American Orthopedic Foot and Ankle Society
AVN: Avascular necrosis
BMI: Body Mass Index
DMAA: Distal metatarsal articular angle
DMO: Double metatarsal osteotomy
FML: First metatarsal length
HV: Hallux valgus
HVA: Hallux valgus angle
IMA: Intermetatarsal angle
RSO: Revolving scarf osteotomy
SD: Standard Deviation
VAS: Visual analog scale
